# Genome-wide detection of positive and balancing signatures of selection shared by four domesticated rainbow trout populations (*Oncorhynchus mykiss)*

**DOI:** 10.1186/s12711-024-00884-9

**Published:** 2024-02-22

**Authors:** Katy Paul, Gwendal Restoux, Florence Phocas

**Affiliations:** Université Paris-Saclay, INRAE, AgroParisTech, GABI, 78350 Jouy-en-Josas, France

## Abstract

**Background:**

Evolutionary processes leave footprints along the genome over time. Highly homozygous regions may correspond to positive selection of favorable alleles, while maintenance of heterozygous regions may be due to balancing selection phenomena. We analyzed data from 176 fish from four disconnected domestic rainbow trout populations that were genotyped using a high-density Axiom Trout genotyping 665K single nucleotide polymorphism array, including 20 from the US and 156 from three French lines. Using methods based on runs of homozygosity and extended haplotype homozygosity, we detected signatures of selection in these four populations.

**Results:**

Nine genomic regions that included 253 genes were identified as being under positive selection in all four populations Most were located on chromosome 2 but also on chromosomes 12, 15, 16, and 20. In addition, four heterozygous regions that contain 29 genes that are putatively under balancing selection were also shared by the four populations. These were located on chromosomes 10, 13, and 19. Regardless of the homozygous or heterozygous nature of the regions, in each region, we detected several genes that are highly conserved among vertebrates due to their critical roles in cellular and nuclear organization, embryonic development, or immunity. We identified new candidate genes involved in rainbow trout fitness, as well as 17 genes that were previously identified to be under positive selection, 10 of which in other fishes (*auts2*, *atp1b3*, *zp4*, *znf135*, *igf-1α*, *brd2*, *col9a2*, *mrap2*, *pbx1*, and *emilin-3*).

**Conclusions:**

Using material from disconnected populations of different origins allowed us to draw a genome-wide map of signatures of positive selection that are shared between these rainbow trout populations, and to identify several regions that are putatively under balancing selection. These results provide a valuable resource for future investigations of the dynamics of genetic diversity and genome evolution during domestication.

**Supplementary Information:**

The online version contains supplementary material available at 10.1186/s12711-024-00884-9.

## Background

Any animal or plant population, wild or domesticated, evolves through continuous and cumulative changes over time [1] that rely on various evolutionary forces, i.e. mutation, migration, selection, and genetic drift, with relative effects that may vary depending on population history and structure. For example, when the effective population size is small, genetic drift is more significant and randomly induces fixation of alleles. This can lead to degeneration and extinction of small populations due to the fixation of deleterious alleles [2]. When environmental conditions change, allele frequencies will change to a new relevant equilibrium as a result of natural selection. Indeed, alleles that are favorable in a particular environment because they carry new mutations or because of standing variation, will be positively selected. In wild populations, favorable alleles generally affect fitness through individual survival, mating, or fertility [3, 4]. Natural selection can also act by negative (or purifying) selection that hinders the spread of deleterious alleles [5]. These two processes tend to reduce genetic diversity at the target genes but have different effects on the genome, with positive selection leading to stronger signatures of selection than negative selection. Conversely, polymorphisms within a population can be actively maintained in some genomic regions through balancing selection that maintains an equilibrium in the frequencies of alleles. The two main biological causes of balancing selection are heterozygote advantage at a single locus, known as the overdominance effect, and frequency-dependent selection, with a rare-allele advantage that tends to restore an equilibrium of the frequencies of alleles at the population level [6, 7].

Domestication is the evolutionary process of genetic adaptation of a wild population to human handling and breeding in captive environments over generations [8–10]. During domestication, humans exert artificial selection pressure by choosing for reproduction the individuals that are most adapted to cohabitation and have aptitudes that best fit their needs [11, 12], such as less fearfulness of humans [13, 14]. Domestication induces severe genetic bottlenecks due to the selection and reproduction of only a few adapted animals from the wild population. Thus, many genetic evolutionary processes have a significant role in the evolution of farmed animal populations, including selection, genetic drift, and inbreeding [15, 16]. Domestication affects life history traits due to changes in morphological, physiological, reproductive, behavioral, and immune functions [16–18] compared to their wild relatives [8, 9]. Wilkins et al. [19] suggested that these specific modifications, called the domestication syndrome, may be due to a mild deficit in neural-crest cells during embryonic development in domesticated animals. In addition, both natural and artificial selection of domesticated populations leave footprints across the genome, known as signatures of selection, which can point to regions that harbor essential genes for domestication or survival [20–22].

Compared to terrestrial animals [16], domestication of fish is recent and was first documented with carp about 2000 years ago [23]. The precise date and location (Neolithic China or during the Roman period in Central and East Europe) of the domestication of carp are still debated [23, 24]. However, most farmed fish species have only been domesticated since the last century. Rainbow trout is native to the Pacific drainages of North America and to Kamchatka in Russia and its domestication started in the 1870s in California [25, 26], and domesticated fish were then introduced in Western Europe at the beginning of the twentieth century [27].

Numerous studies have been carried out over the last ten years to detect signatures of selection in farmed fish species (channel catfish [28]; Atlantic salmon [29–33]; carp [34]; Nile tilapia [35–37]; rainbow trout [38]; Coho salmon [39]; Australasian snapper [40]; brown trout [41]) in order to identify genomic regions that are involved in recent adaptation or domestication processes [42, 43]. Various approaches have been developed to detect signatures of selection within populations based on site frequency spectrum, linkage disequilibrium (LD), or reduction in local variability [44, 45]. Among these approaches, we used two strategies, one based on the reduction of local variability using runs of homozygosity (ROH) metrics and the second based on allele frequencies and the extent of LD, using extended haplotype homozygosity (EHH). A ROH is a long homozygous stretch in the genome of an individual that is putatively homozygous by descent and thus inherited from a common ancestor to its parents [46, 47], while EHH measures the extent of shared haplotypes through the association between a single core haplotype and multiple loci at various distances from the core region [48].

In this study, we were interested in the identification of genes that have been under either positive or balancing selection in farmed rainbow trout populations, since this species is one of the oldest domesticated fish. The wild populations present in the McCloud River basin of North California are thought to be the origins of all strains that are currently domesticated [49, 50]. Early domestication events were traced back to the 1870s and France was one of the first countries that imported domesticated strains [51]. Only a few studies on signatures of selection have been performed in rainbow trout, of which three focused on wild populations and showed signatures of selection that were linked to life-history variation, egg development, spawning time [52], immune response [53], and smoltification [54]. The first study in domesticated rainbow trout was performed on a single Chilean population [38] that was genotyped with a 57K single nucleotide polymorphism (SNP) array and showed that the identified signatures of selection were associated with early development, growth, reproduction, and the immune system. Recently, a high-density array (665K single nucleotide polymorphisms (SNPs)) was developed for rainbow trout [55], allowing us to potentially detect signatures of selection more accurately and to compare them across various domesticated rainbow trout populations. The presence of signatures of selection that are shared by multiple disconnected farmed populations from different geographical areas allows the identification of genomic regions that are effectively linked to the domestication process or to fitness and thus avoids identification of local signatures that are present only in some populations. This approach will help to better understand the genomic processes at play and the subsequent dynamics of genetic diversity in rainbow trout, by identifying genes that have key roles in either the domestication process or in fitness [56, 57].

In this study, we considered four populations: one INRAE experimental line (with no intentional selection) that displays a wide diversity due to its multiple origins, two French selection lines each from a different breeding company, and a pooled American population that includes samples from one wild river and four hatchery strains, all from the North-West of the USA and closely linked genetically [58]. The availability of such a variety of origins should allow us to detect, genomic regions for which it was biologically important that they remained either homozygous or heterozygous, independent of the history of the investigated strains. This is also why we chose a North-American population rather than a Californian one.

The aim of our work was to discover genomic regions with a high level of homozygosity (positive selection) or heterozygosity (balancing selection) that are shared across the four rainbow trout populations and to get further insights into the nature of the genes that span these regions.

## Methods

### Populations

Three French populations were considered: 14 breeding females from the INRAE synthetic line SY and, 90 and 72 females from selection lines LB and LC from the breeding companies “Bretagne Truite” (Plouigneau, France) and “Viviers de Sarrance” (Sarrance, France) respectively. As described by D’Ambrosio et al. [59], the two commercial lines were selected on individual growth by optimized within-group mass selection and on carcass traits based on sib testing. Genotyped females from the LB and LC lines (previously described as SB and SC in [59]) were breeders from the 8th and 10th generations of their respective selected lines. The SY line was developed by intercrossing several domesticated lines of rainbow trout, in order to create a population with a large diversity [59].

**Fig. 1:**
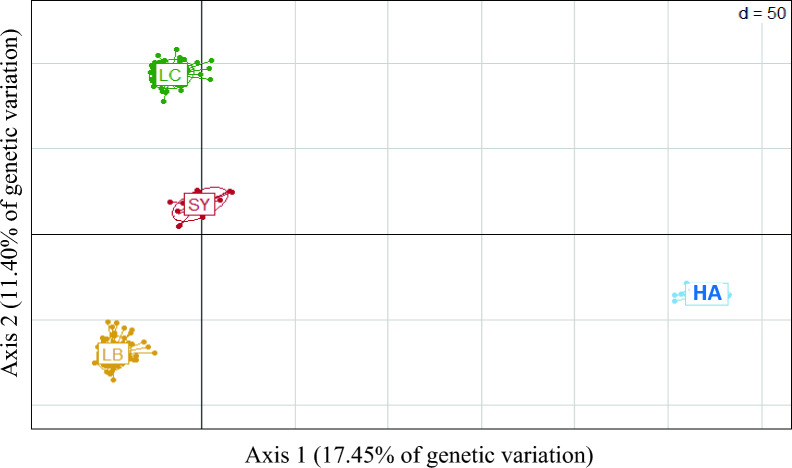
Principal component analysis (PCA) of the genetic diversity of the five North American subpopulations from the HA population based on 546,903 SNPs. Elwha is the only wild population

We also analyzed the whole-genome sequence data from 20 fishes from a pooled population of American trout described by Gao et al. [58]. The 20 fish included four individuals at each of five locations from the North-West of the USA: wild fish from Elwha River, and farmed fish from Dworshak, L. Quinault, Quinault, and Skamania hatcheries. We pooled these 20 individuals together, as these five populations were genetically close to each other (see Additional file 1: Fig. S1a; [58]) and very distant from the three French populations (Fig. 1).

### Genotyping and quality control

High-density genotypes were obtained at the INRAE genotyping platform Gentyane (Clermont-Ferrand, France) for the 176 French samples using the Affymetrix 665K SNP array that was recently developed for rainbow trout [55]. We considered only the genotypes for the 576,118 SNPs of the Rainbow trout Axiom® 665K SNP array that were positioned on the Arlee reference genome (GCA_013265735.3, [55, 60]). From the whole-genome sequence information of the 20 American samples [58], we extracted the genotypes for the same 576,118 SNPs. Among the 176 French genotyped fish, 19 individuals with more than 30% identity-by-state (IBS) with other individuals were removed from the dataset, leaving 76, 67, 20, and 14 fish from the LB, LC, HA, and SY populations, respectively.

SNP quality control was performed using the PLINK v1.9 software [61]. To avoid limitations due to the small number of individuals in the SY population, quality filters were applied to the LC and SY individuals together, as both these populations were genotyped on the same SNP plate and are genetically close [59]. SNPs with a call rate lower than 97% were removed as well as about 4000 SNPs randomly distributed along the genome due to extreme deviations from Hardy–Weinberg equilibrium (p-value < 10^–7^). This allowed us to discard SNPs with a high risk of genotyping errors and a low probability of being under selection. We retained 571,319, 569,030, and 573,793 SNPs for the LB,’LC- SY’, and HA populations, respectively. Finally, by merging the three SNP lists, we kept the 546,903 common SNPs for further analysis.

### Genetic structure of the populations

Genetic differentiation between the populations was measured using the pairwise Fst estimate in the VCFtools v0.1.13 software [62]. In addition, a principal component analysis (PCA) was performed with the R package *Adegenet* (function *glPca*) [63] to visualize the genetic structure of the populations.

### Runs of homozygosity

Runs of homozygosity (ROH) were identified for each fish using the PLINK v1.9 *homozyg* function [61] with the following options *‘–homozyg-kb 500 –homozyg-window-snp 40 –homozyg-snp 40 –homozyg-gap 500 –homozyg-density 40 –homozyg-het 1*’. A ROH was defined by a sliding window with a minimum length of 500 kb and at least 40 homozygous SNPs. This minimum number of homozygous SNPs was chosen using the formula described by Purfield et al. [47] in order to limit the number of ROH that can occur by chance. Up to one possible heterozygous genotype was permitted for each ROH.

### Estimation of inbreeding coefficients

Individual inbreeding coefficients (${F}_{ROH}$) were calculated according to McQuillan et al. [46] as ${F}_{i,ROH}=\frac{\sum length({ROH}_{i})}{LGenome}$, where $\sum length({ROH}_{i})$ is the sum of the lengths of ROH for individual $i$ and $LGenome$ is the total length of the autosomal genome covered by SNPs.

### Identification of ROH islands

For each SNP, the number of individuals with this SNP included in a ROH was calculated in order to identify regions of the genome that were frequently homozygous in each population, i.e. constituting ROH islands [64], which can be considered as signatures of positive selection [65]. To allow for comparison of ROH islands across populations, we implemented population-specific thresholds based on the number of individuals with a ROH to define ROH islands, as proposed in many studies, with an empirical SNP-based threshold for each population equal to the number of individuals with a ROH observed for the top 5% of SNPs, rather than the top 1% that is used in many studies [66–72]. The use of a less stringent threshold was because our objective was to search for common regions across populations. These top 5% values were equivalent to 35, 27, 5, and 10 individuals for the LB, LC, SY, and HA populations, respectively, which correspond to 48.6, 40.3, 35.7, and 50% of the individuals with a ROH in the LB, LC, SY, and HA populations, respectively. In addition, SNPs in the top 5% that were less than 500 kb apart were considered to fall in the same ROH island if the number of SNPs in the gap stretch between two SNPs of the top 5% was less than 40. The boundaries of a ROH island were defined so that a minimum of 30, 22, 3, and 7 individuals (thresholds for the top 10% of SNPs) were homozygous for any SNP within the ROH for the LB, LC, SY, and HA populations, respectively.

### Detection of balancing selection signals based on regions without ROH

We used the information on ROH occurrence per SNP to detect extreme heterozygous regions, i.e. without ROH. These regions have an enrichment of heterozygous SNPs relative to the genome-wide prevalence, which may be due to balancing selection [73].

Applying the same criteria as used to define ROH, the minimum size and number of SNPs to define a heterozygous region were fixed to 500 kb and 40 SNPs, respectively. Moreover, two successive SNPs were considered in the same heterozygous region if they were separated by less than 50 kb. A region was defined as having an extreme level of heterozygosity (i.e., hotspot of polymorphism) if less than 5% of the individuals (per population) had SNPs in the ROH within the region, corresponding to a maximum of four and three individuals with a ROH in the LB and LC populations, respectively, and no individuals with a ROH in the SY and HA populations.

### Detection of signatures of selection based on extended haplotype homozygosity (EHH)

For a given focal allele, the EHH is defined as the probability that two randomly chosen chromosomes that carry the core haplotype of interest are identical-by-descent for the entire interval from the focal locus to the locus at distance x [48]. EHH measures the association between a single allele at the focal locus with multiple loci at various distances x from the focal locus [48]. The integrated haplotype homozygosity score (iHS) proposed by Voight et al. [74] compares the integrated EHH profiles obtained for a SNP in the ancestral versus derived states. An extreme value for iHS corresponds to positive selection because a focal haplotype with an unusually high EHH and a high frequency in the population indicates the presence of a mutation that has spread through the population at a faster rate than disruption of haplotypes by recombination.

Because the EHH methodology requires haplotype information, the genotype data must be phased before their calculation. We used FImpute3 [75] to phase the genotypes of the fish under study, by considering all the parents and offspring that were genotyped in the LB, LC, and SY populations for different purposes (see respectively [76–78]. All parents of the evaluated fish (except eight SY sires) were genotyped with the HD chip [55]**,** while offspring (and eight SY sires) were genotyped with a 57K chip [79]. The information used for phasing is in Table 1. Due to the lack of genotyped offspring, only the HD genotypes were used to phase the genotypes of the HA population.

**Table 1:** Numbers of individuals and SNPs available after quality control that were used to phase the HD genotypes of the females under study that belonged to parental cohorts

Line	Status of the individuals	Number of individuals	Number of SNPs used
LB	Parents	288	571,319
	Offspring	1297	29,091
LC	Parents	173	569,03
	Offspring	1350	30,379
SY	Parents (dams + 1 sire)	16	569,03
	Offspring (+ 8 sires)	866	32,725

Once phasing was performed, the *rehh* R package [80, 81] was used to conduct EHH-based analyses. Detection of EHH was stopped when the EHH value was lower than 0.1 or when the gap between two consecutive SNPs was larger than 20 kb (*scan_hh* function with the following options: limehh = 0.1; maxgap = 20 kb).

### Cross-population extended haplotype homozygosity (XP-EHH)

From the EHH information, we used the XP-EHH statistics (*ies2xpehh* function) to compare the integrated EHH profiles (iES) between a French (popA) and the HA (popB) populations at a focal SNP [82] as:

$${X{P}}\_{EHH}=\frac{{\text{ln}}\left(\frac{{iES}_{popA}}{{iES}_{popB}}\right) -Med\left[{\text{ln}}\left(\frac{{iES}_{popA}}{{iES}_{popB}}\right)\right]}{SD \left[{\text{ln}}\left(\frac{{iES}_{popA}}{{iES}_{popB}}\right)\right]}.$$

where $Med$ is the median and $SD$ is the standard deviation of $\frac{{iES}_{popA}}{{iES}_{popB}}$, which were computed across all analysed SNPs.

### Integrated haplotype homozygosity score (iHS)

We used the iHS test [74] to evaluate evidence of positive selection based on haplotype frequencies in a single population, using the *ihh2ihs* function of the package *rehh*. This statistic is based on the log-ratio of the integrated EHH (iHH) for haplotypes with the ancestral ($A$) *versus* the derived ($D$) alleles and was computed for each autosomal SNP as $iHS=\frac{{\text{ln}}\left(\frac{{iHH}_{A}}{{iHH}_{D}}\right) - {Mean}_{p}\left[{\text{ln}}\left(\frac{{iHH}_{A}}{{iHH}_{D}}\right)\right]}{{SD}_{p}\left[{\text{ln}}\left(\frac{{iHH}_{A}}{{iHH}_{D}}\right)\right]}$, where ${Mean}_{p}$ is the average and (${SD}_{p}$ is the standard deviation of ${\text{ln}}\left(\frac{{iHH}_{A}}{{iHH}_{D}}\right)$, which were computed across all the SNPs with a derived allele frequency $p$ similar to that of the focal SNP. Since the state of the ancestral allele was unknown in our study, we arbitrarily assumed that the most frequent allele represents the ancestral state, as proposed by Bahbahani et al. [83]. This assumption is likely valid for neutral loci or for loci with mutant alleles that are under negative selection compared to the common wild type alleles. However, for domesticated or selected populations, the ancestral state is expected to be the most frequent allele under positive selection. However, as the iHS values are normally distributed [83], a two-tailed Z-test was applied to identify statistically significant SNPs under selection with an extended haplotype of either the ancestral (positive iHS value) or the derived alleles (negative iHS value). We then arbitrarily considered that any extreme iHS absolute value (i.e. |iHS|≥ 2.5) corresponds to a positive signature of selection either for the ancestral allele (iHs > 0) or for the derived allele (iHs < 0).

### Detection of candidate regions

To detect candidate regions for signatures of selection based on the iHS test, we used *the calc_candidate_region* function of the R package *rehh* [80]. We considered sliding windows of 500 kb along the genome that contained at least 30 SNPs and that overlapped by 10 kb. A region was considered to have been under positive selection if at least one SNP had a − log(p-value) > 4 and an extreme iHS value, i.e. |iHS|≥ 2.5.

### Identification of shared regions under positive selection

ROH islands and regions identified by iHS were pooled within each population and regions that were identified by either method for all four populations were identified. We eliminated regions for which one population did not have at least one SNP with an |iHS|≥ 2.5 or enough individuals with a ROH in the shared region. Thus, only regions that contained either ROH islands or an extreme iHS (|iHS|≥ 2.5) for each of the four populations were further analyzed.

### Gene analysis

Annotated genes in the regions under positive or balancing selection were identified from the NCBI *Oncorhynchus mykiss* genome assembly (GCA_013265735.3). Gene symbols were checked, and, if necessary, commonly used names were added using the information available from GeneCards (https://www.genecards.org/). A study of gene ontology (GO) terms was performed for the list of genes identified in the regions of interest using the 'g:profiler' web server ([84]; https://biit.cs.ut.ee/gprofiler/gost). The percent identity between corresponding proteins (of any annotated gene) for rainbow trout and nine other vertebrate species (human, mouse, cow, goat, pig, chicken, zebrafish, medaka, and Atlantic salmon) was established using the blastp tool (local alignment search tool on proteins in NCBI platform and protein alignments were obtained from NCBI).

## Results

### Genetic diversity within and across populations

The ROH statistics and inbreeding coefficients for the four populations are in Table 2. The average number of ROH per individual ranged from 141 (SY) to 168 (LB). The average size of the ROH was larger for the French selected lines than for the SY and HA populations. The average inbreeding coefficients of the HA individuals were between three (compared to SY) and five (compared to LB) times lower than those of the French lines. Within the pooled HA population, the average inbreeding coefficient was very low (F_ROH_ = 0.02) for the wild HA sub-population and ranged from 0.04 to 0.06 for the other HA sub-populations.

**Table 2:** ROH statistics and inbreeding coefficients (F_ROH_) of the four populations

Population	Average number of ROH	Average size of ROH (in kb)	Average F_ROH_
LB	168 (14.6)	2770 (270.8)	0.20 (0.02)
LC	157 (15.9)	2485 (326.8)	0.17 (0.03)
SY	141 (33.5)	1860 (291.2)	0.12 (0.05)
HA	167 (65.6)	1433 (145.6)	0.04 (0.03)

Standard deviations are between brackets

Based on genome-wide Fst, a large differentiation (~ 0.289) was observed between HA and any of the French populations (Table 3). In the PCA figure (Fig. 1), the three French lines were strongly differentiated from the American pooled populations, and the first two PCA axes explained 29% of the total genetic variation. In addition, Fst indicated that all the French lines were moderately differentiated (0.104 to 0.122). Among the five HA sub-populations, those from L. Quinault, Quinault, and Elwha river were very close to each other (0.01 < Fst < 0.03), while their genetic differentiation was larger, but weak, with fish from the Skamania hatchery (0.03 ≤ Fst ≤ 0.05). It is worth noting that fish from the Dworshak hatchery diverged the most from the others and not the group of wild fish (from the Elwha river) (see Additional file 1: Fig. S1b).

**Table 3:** Genome-wide Fst between the four populations

	LC	LB	HA
SY	0.104	0.122	0.275
LC		0.121	0.274
LB			0.289

Using the XP-EHH statistics, we identified 93, 105, and 135 regions that strongly discriminated the HA from the LB, LC, and SY populations, respectively. Among these regions, 34 were shared, spanned about 32 Mb across 21 chromosomes, and differentiated all the French lines from the American HA pooled population (see Additional file 2: Table S1).

**Fig. 2:**
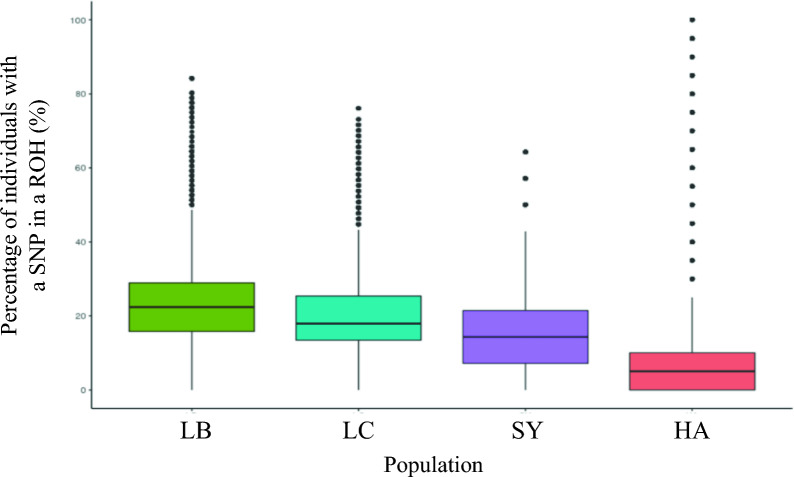
Box-plots of the occurrence of ROH (number of individuals with this ROH) per SNP for the four rainbow trout populations, LB, LC, SY, and HA

The distribution of the proportion of individuals having a ROH at each SNP position is in Fig. 2. On average, more ROH were shared between individuals within the selected lines (LB and LC, on average 23.4% and 19.8% of individuals, respectively) than for other populations (SY and HA, on average 13.7% and 8.9% of individuals, respectively). The HA population showed the smallest number of shared ROH among individuals, which is probably a result of its composite nature (5 sub-groups of 4 individuals), but it had also the largest number of individuals that shared a given ROH.

### Signatures of positive selection

#### ROH islands

We listed all ROH islands within each population and identified 270 ROH islands distributed among the four populations (see Additional file 2: Tables S2, S3, S4, and S5 for the LB, LC, SY, and HA populations, respectively). The ROH islands were not evenly distributed across populations and chromosomes. The average ROH island size was 2737 kb, ranging from 1593 to 4465 kb, depending on the population. The longest (21.4 Mb) and shortest (16.1 kb) ROH islands were observed for the SY and LC populations, respectively.

**Fig. 3:**
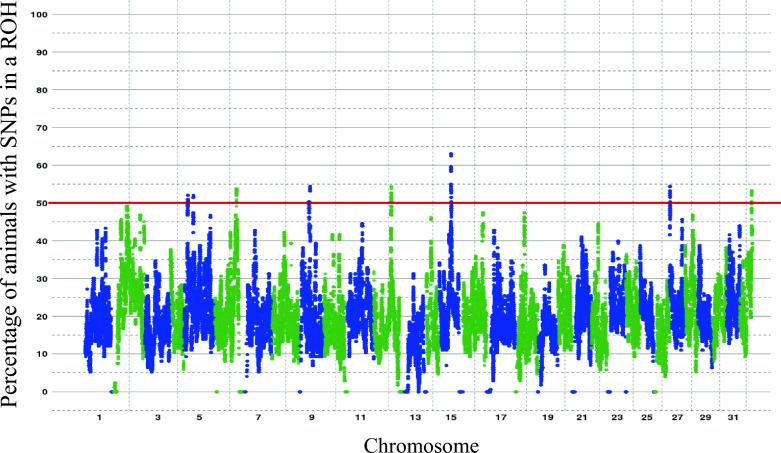
Manhattan plot of the occurrence of ROH per SNP across chromosomes (across the four rainbow trout populations). The red line corresponds to the threshold of 50% selected to consider a region as a ROH island

The shared ROH among individuals are presented in Fig. 3. Eight ROH islands were shared by at least two populations, with a minimum of 50% individuals involved for each population. However, only three of these regions with ROH were defined as ROH islands in all four populations.

#### Signatures of selection based on iHS

**Fig. 4:**
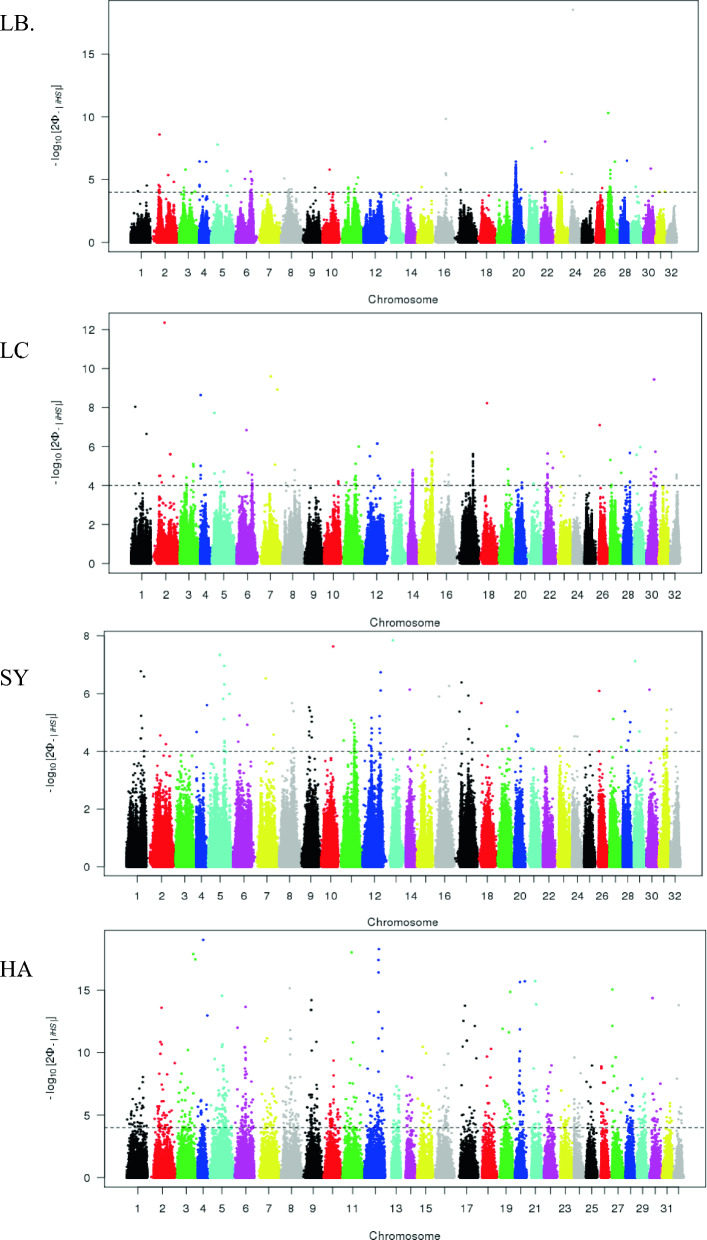
Genome-wide distribution of -log(p-value) for standardized iHS for each of the four rainbow trout populations (LB, LC, SY, HA). The dashed line indicates the -log(p-value) significance threshold set to 4 to identify regions under positive selection

The − log(p-values) of the iHS calculated across the genome are presented in Fig. 4 for each population (all regions identified with *calc-candiate_region* are described in Additional file 2: Tables S6, S7, S8 and S9. The genome-wide highest estimates of |iHS| were 8.97, 7.24, 5.67, and 9.09 for the LB, LC, SY, and HA populations, respectively (with − log(p-values) > 7.8). While numerous regions were identified to be under positive selection overall, fewer such regions were identified for the French lines (LB, LC and, SY) than for the American pooled population (HA). This may be the result of the pooled origins of the HA population which can produce false positive signatures of selection. Because our objective was not to detect signatures of selection within populations but across populations, the shared signatures of selection are expected to be robust to any bias that could be due to the structure of the HA population.

**Fig. 5:**
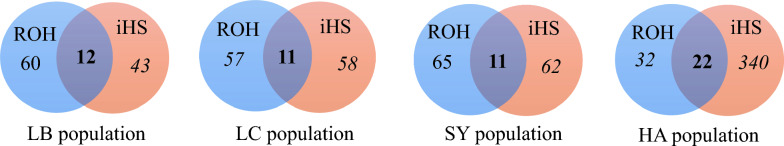
Venn diagram of the number of regions identified as ROH island or iHS signatures of selection for each of the four rainbow trout populations

In total, 72, 68, 76, and 54 ROH islands were identified for the LB, LC, SY, and HA populations, respectively (Fig. 5). Using iHS statistics, 55, 69, 73, and 362 signatures of selection were detected for the LB, LC, SY, and HA populations, respectively. Only 10.4, 8.7, 8.0, and 5.6% of the regions were detected by both methods (ROH and iHS) for the LB, LC, SY, and HA populations, respectively.

#### Regions under positive selection shared by the four populations

Among the numerous regions identified for each population by either the ROH or the iHS method, only nine regions were shared by the four populations (Table 4). The average size of these shared regions was 1135 kb. Among these nine regions, five were located on chromosome 2, and the other four were on chromosomes 12, 15, 16, and 20.

**Table 4:** Homozygous regions under positive selection in the four populations

Region	CHR	Start (Mb)	End (Mb)	Size (kb)
chr2_a	2	25.40	26.30	900
chr2_b	2	31.60	34.20	2600
chr2_c	2	46.00	46.66	664
chr2_d	2	69.70	71.20	1500
chr2_e	2	88.46	89.34	878
chr12_a	12	57.97	59.10	1138
chr15_a	15	38.96	39.57	610
chr16_a	16	45.80	47.00	1200
chr20_a	20	19.10	19.83	726

Two regions, chr2_c and chr15_a (Table 4), were detected only based on the ROH analysis in each population, while chr16_a was identified only based on the significant iHS statistics in each population (see Additional file 2: Table S10). The list of genes annotated in the nine shared genomic regions is in Additional file 2 Table S11.

### Signatures of balancing selection

#### Regions under balancing selection detected within population

In total, 14, 24, 158, and 265 hotspots of polymorphism (i.e. without ROH) were identified for the LB, LC, SY, and HA populations, respectively. The numbers of heterozygous regions detected were substantially larger for the SY and HA populations than for the selected lines LB and LC. The average size of the detected heterozygous regions was 1400 kb, ranging from 1086 to 1828 kb, depending on the population. Additional file 2: Tables S13 to S16 list all heterozygous regions within each population.

#### Regions under balancing selection shared by the four populations

A substantial lack of ROH was observed for each population (Table 5) in four regions. Two of these regions, chr10_a and chr19_a (Table 5), were particularly small (53 kb and 70 kb, respectively), but still contained at least 20 SNPs. The chr10_a region encodes one of the introns of the *ctnna2* (*catenin alpha 2*) gene, while the chr19_a region covered two genes, *smarca5* (*SWI/SNF-related matrix-associated actin-dependent regulator of chromatin subfamily A member 5*) and *frem2* (*FRAS1-related extracellular matrix protein 2*). A second heterozygous region on chromosome 19 was larger (163 kb) and contained one annotated gene *pou4f2* (*POU domain, class 4, transcription factor 2-like*). The fourth region, chr13_a, spanned over 1100 kb on chromosome 13 and included 25 genes. The list of genes annotated in the four shared genomic regions is in Additional file 2: Table S17.

**Table 5:** Highly heterozygous regions shared by the four populations

Region	CHR	Start (Mb)	End (Mb)	Size (kb)	SNP number	SNP density per Mb
chr10_a	10	56.314	56.366	53	20	379
chr13_a	13	46.959	48.071	1112	446	401
chr19_a	19	10.753	10.823	70	24	342
chr19_b	19	11.354	11.517	163	52	319

### Identification and role of the genes in the regions under selection across all populations

#### Homozygous regions under positive selection shared by the four populations

The nine homozygous regions that were common to the four populations contained 253 genes (see Additional file 2: Table S11). The GO study showed significant over-representation (*p-value* < *0.01*) of genes with functions related to the following GO terms: membrane (GO:0016020, CC: cellular component*, p-value* = *1.3e10*^*−5*^), intrinsic and integral component of membrane (GO:0031224; GO:0016021, CC, *p-value* = *0.001/0.005*), ion binding (GO:0043167, MF: molecular function*, p-value* = *0.002*), and nuclear speck (GO:0016607, CC*, p-value* = *0.008*).

Among these nine regions, the regions chr2_a, chr2_c, and chr15_a, which each contained less than 10 annotated genes, were analyzed in further detail to accurately define the roles of these genes. The 17 genes located in these three regions are listed in Additional file 2: Table S12, with their associated biological functions that play key roles in protein transduction/maturation, genome stability, embryonic development, growth, energetic function, reproduction, or immune function [85–114]. Detailed information for 15 genes in six other homozygous regions that were previously identified as signatures of selection [30, 33, 38, 40, 65, 115–122] is also provided in Additional file 2: Table S12.

We estimated the degree of protein identity among 10 vertebrate species for the 17 genes in the chr2_a, chr2_c, and chr15_a regions (Table 6), considering a protein as highly conserved if its identity between rainbow trout and other species was higher than 85%. Except for the proteins encoded by the *cep162* (*centrosomal protein of 162 kDa*) and *zp4* (*zona pellucida sperm-binding protein 4-like*) genes, all the other proteins were at least highly conserved between the two studied salmonids.

**Table 6:** Percentage of protein identity between rainbow trout and nine other vertebrate species for all the genes annotated in the homozygous regions chr2_a, chr2_c and chr15_a

Region	gene_ID	Human	Mouse	Goat	Cattle	Pig	Chicken	Zebrafish	Medaka	Atlantic salmon
chr2_a	mrap2a	45.71	43.52	43.87	44.98	45.45	42.20	56.22	50.45	87.55
	cep162	36.61	37.50	40.95	40.95	50.00	53.73	38.61	62.50	80.66
	adgrb1	62.37	63.44	62.12	61.95	61.74	67.72	84.02	80.94	98.16
	tsnare1	54.78	31.50	56.99	55.79	56.02	60.74	85.62	78.55	98.29
	pttg1IP	60.00	57.89	57.04	57.04	58.82	59.74	70.92	66.03	93.89
	cdk14	87.05	87.05	86.44	86.02	85.99	87.24	88.96	91.08	99.58
chr2_c	brsk2a	92.12	92.50	92.66	92.19	92.66	93.82	96.14	92.05	96.26
	abtb2b	71.54	70.76	71.93	71.74	71.74	72.46	81.05	61.68	97.35
	b4galnt4a	63.35	65.38	57.14	64.63	64.95	66.37	66.06	79.96	96.55
chr15_a	chn1	88.80	86.59	88.04	87.32	88.04	88.10	85.29	85.01	98.04
	atp5mc1	97.37	87.10	91.76	94.44	93.33	86.17	91.30	97.87	94.12
	zc3h15	69.35	68.57	67.55	67.55	67.55	66.90	74.33	71.57	97.30
	zp4	29.67	29.61	31.87	37.47	30.21	31.46	45.60	49.74	74.74
	nid2	52.00	51.16	51.11	51.11	51.22	55.00	58.14	52.78	97.87
	brca2	46.24	43.72	37.12	32.42	45.61	45.85	38.68	54.08	92.05

In each of the three regions, one or two genes were highly conserved across the 10 studied species. For the chr2_a region, the protein encoded by the rainbow trout *cdk14* (*cyclin-dependent kinase 14*) gene had a percent identity between 86 and 99.6% with the other species; for the chr2_c region, the protein encoded by the rainbow trout *brsk2a (serine/threonine-protein kinase brsk2*) gene had between 92 and 96.3% identity with the other species; and for the chr15_a region, the proteins encoded by the two *chn1*(*n-chimaerin*) and *atp5mc1* (*ATP synthase lipid-binding protein, mitochondrial*) genes also had a protein percent identity ranging from 85 to 98% depending on the species.

Two other rainbow trout proteins encoded by the *tsnare1* (*t-SNARE domain-containing protein 1*) and *pttg1IP* (*pituitary tumor-transforming gene 1 protein-interacting protein*) genes were conserved to a lesser extent (minimum 65% identity) with the three other fish species, several were also conserved at least with chicken *adgrb1* (*adhesion G protein-coupled receptor B1*), *b4galnt4a* (*N-acetyl-beta-glucosaminyl-glycoprotein 4-beta-N-acetylgalactosaminyltransferase 1*) or even with all nine studied species *zc3h15* (*zinc finger CCCH domain-containing protein 15*).

#### Heterozygous regions under balancing selection shared by the four populations

The four heterozygous regions (Table 5) that were common to the four populations contained 29 genes (see Additional file 1: Table S17). A study of GO terms showed no significant over-representation of specific GO terms. The degrees of protein % identity among the 10 vertebrate species for these 29 genes are in Additional file 2: Table S18.

The three regions chr10_a, chr19_a, and chr19_b contained only a few genes and their analysis (Table 7) did not show any key roles in cellular and nuclear organisation and in embryonic development.

**Table 7:** List and functions of the four genes annotated in three heterozygous regions (chr10_a, chr19_a, and chr19_b) shared by the four populations

Region	Gene name	Protein name	General function	References
chr10_a	*ctnna2*	catenin alpha 2	Enables actin filament binding activity, and involved in negative regulation of Arp2/3 complex-mediated actin nucleation. Regulation of neuron migration and of neuron projection development. Implicated in brain malformations. Seems implicated in vertebral development(/deformities) in Yunlong grouper	[123] vertebrates; [124] Yunlong grouper
chr19_a	*smarca5*	SWI/SNF-related matrix-associated actin-dependent regulator of chromatin subfamily A member 5	The protein encoded by this gene is a member of the SWI/SNF family of proteins. Members of this family have helicase and ATPase activities and are thought to regulate transcription of certain genes by altering the chromatin structure around those genes. The protein encoded by this gene is a component of the chromatin remodeling and spacing factor RSF, a facilitator of the transcription of class II genes by RNA polymerase II	[125] zebrafish; [126] mice: [127] zebrafish
	*frem2*	FRAS1-related extracellular matrix protein 2	Plays a role in epidermal-dermal interactions—> important for the integrity of skin and renal epithelia	[128] zebrafish
chr19_b	*pou4f2*	POU domain, class 4, transcription factor 2-like	May be involved in maintaining visual system neurons in the retina, and in the lateral line. The level of the encoded protein is also elevated in a majority of breast cancers, resulting in accelerated tumor growth. Seems link to cardiac development in zerafish	[129] zebrafish; [130] zebrafish

## Discussion

The objective of our study was to detect signatures of selection in domestic rainbow trout. To reach that goal, we studied four genetically distinct populations that were sampled from different locations in France and in the North-West of the USA. Using two approaches, ROH and EHH, and genotypes from the 665K SNP array, we detected nine very conserved regions and four hotspots of polymorphism, which included 253 and 29 annotated genes, respectively.

### Quality of the 665 K SNP array

This HD array has been carefully designed to filter out putatively duplicate markers [55] that are due to the complexity of the rainbow trout genome, which results from the fourth whole-genome duplication event that occurred about 96 Mya [131], leading to only partial rediploidization, and patterns of tetrasomic inheritance [132]. These phenomena may explain the difficulties to sequence and assemble some parts of the rainbow trout genome and thus to detect SNPs. Although the 665K SNP chip was designed based on the Swanson reference genome [132], only the 576K SNPs that were uniquely positioned on the Arlee reference genome [60] were considered in our study. A large gap of 2.75 Mb between two subsequent SNPs on the HD array is present at the end of the *Oncorhynchus mykiss* (Omy) chromosome 13 on the Arlee reference genome [55]. This gap is likely due to the fact that Omy13 shares a high level of sequence homology with other chromosome arms due to delay in re-diploidization [58]. The large (> 1.1 Mb) heterozygous region that we identified on the q arm of Omy13 was not located in the telomeric region, but rather 10 Mb towards the centromere in a region that shares homology with the q arm of Omy12, based on the Swanson reference assembly [132]. A high percent identity between chromosome arms can lead to chimerism or assembly collapse, which can give an appearance of excess heterozygosity if not detected. We hypothesize that the new Arlee reference genome assembly allowed us to identify variants from those two homolog regions without ambiguity. For the same reason, it is also important to note that the regions identified as very heterozygous in the centromeric region of Omy10 and on the p arm of Omy12 are highly homologous regions [132].

### Genetic structure

In the Results section, we first described the genetic structure of the studied populations. The three French lines were moderately differentiated, with Fst ranging from 0.10 to 0.12, consistent with estimates by D’Ambrosio et al. [59], which ranged from 0.09 to 0.14 for the same populations but using a 57K SNP array. These moderate differences between the three French populations were consistent with the PCA that we performed and the history of these populations with a partially common INRAE origin [59]. This trend is shared between European farmed populations with, e.g., an average Fst of 0.13 between 12 European rainbow trout strains [133]. Similarly, US farmed rainbow trout populations are also weakly to moderately differentiated, with an average Fst of 0.09 [134] or 0.13 [135] and pairwise Fst ranging from 0.06 to 0.16. We observed a similar pattern in the present study, with individuals from the HA population, which consisted of samples from five locations clustering together in the PCA. However, we observed large differentiation between the French and the US populations, as shown by the high Fst (0.27 to 0.29), which is likely the result of numerous factors, including selection, genetic drift, founder effect and absence of gene flow between these geographically distant populations. In addition, the European farmed populations originated from Californian domesticated strains, which have been shown to differ from strains of North-Western USA [136]. We found 34 haplotypes distributed over 21 chromosomes that differed between the American pooled population (HA) and the three French populations (see Additional file 1: Fig. S1a).

Due to the moderate to large differentiation between the four populations, conserved genomic regions are expected to result from ancient natural selection traces. As the ROH-based inbreeding coefficients were quite high in the French populations (0.12 to 0.20), focusing only on French populations may affect the detection of shared regions that are putatively under selection because of genetic drift or inbreeding. Including unrelated and weakly inbred American fish in this comparative study strengthened the ability to detect regions that are putatively shared at the species level, which is of great interest for understanding genome evolution. Similarly, the mixed nature of the HA population provided better insight into the genetic diversity in rainbow trout and highlighted the importance of the conserved regions.

### Comparison of methods to detect shared signatures of positive selection

We used a double-check of positive selection traces in the genome by using both the ROH and EHH approaches. Regions that are detected by more than one method provide stronger evidence of signatures of selection by reducing the number of false positives [38, 45]. However, for each population, only a few regions were identified by both methods. Although both the ROH and iHS approaches evaluate large homozygous stretches in the genome, iHS considers additional information using haplotypic version and linkage disequilibrium from a focal SNP. The ROH approach detects homozygous regions regardless of their haplotypic versions, contrary to the iHS approach. Thus, the latter may detect a signature of positive selection even if various haplotypes are present at the homozygous state in the population. As a result, the iHS approach can detect recent signatures of selection (before fixation of the favorable alleles), while the ROH-based approach may be more efficient in detecting older signatures of selection [45]. In addition, while the ROH approach only detects large homozygous stretches (at least 500 kb in the present study), iHS can detect small regions under positive selection since the only limit on the size of an EHH region is based on a threshold for a minimum LD (0.10). Consequently, the sizes of the detected homozygous regions ranged from 1065 to 2857 kb based on the ROH approach and from 1000 to 1600 kb based on the iHS approach.

The large number of regions (55, 69, 73, and 362 for LB, LC, SY, and HA, respectively) detected by the iHS approach in our study is consistent with numbers that have been reported for Atlantic salmon [33] or cattle [65], although these studies used a less stringent significance threshold than we did (− log(p-value) $\ge$ 3 and 2, respectively, vs > 4 in our study). Smaller numbers of regions were detected using iHS by Cádiz et al. [38] in rainbow trout and by López et al. [39] in Coho salmon, likely because of the lower density of SNPs that was used in both studies (57K and 200K versus 665K in our study) and the subsequent lower ability to detect LD and haplotypes at a fine scale.

In the Chilean rainbow trout study [38] based on the 5K array, only one signal of positive selection was detected by the iHS approach, which was located at 6.398–14.936 Mb on Omy20 of the Swanson reference genome, which corresponds to the region from 7.488 to 16.111 Mb on Omy20 of the Arlee reference genome. We also detected signals of selection by iHS in each of our four populations, located at 10.5–16.5 Mb for LB, at 11.2–13.3 Mb for LC, at 13.0–14.2 Mb for SY, and at 12.3–13.2 Mb for HA (see Additional file 2: Tables S6–S9, for LB, LC, SY, and HA, respectively). Thus, all these signals were consistent with the larger region identified by Cádiz et al. [38]. We identified a putative signature of selection that was shared by all four populations that was located at 13.0–13.2 Mb on Omy20. In this 200-kb-region, we observed at least one iHS value higher than |2.5| for the LB, LC, and SY lines, but not for the HA population. Six annotated genes were identified in this region (*lgi1*, *noc3l*, *plce1*, *slc35g1*, *fra10ac1*, and *tbc1d12*). Two of these genes, were reported as candidates genes associated with domestication by Cádiz et al. [38], *noc3l* and *plce1*. These two genes are also related to early development traits in zebrafish (*noc3l* [137] and *plce1* [138]).

### Biological functions of genes under positive or balancing selection

Most of the 282 genes in the 13 regions that were detected under either positive or balancing selection, are assumed to play essential roles in major biological functions (genome stability, cell organization, neuronal and embryonic development, energy metabolism, growth, reproduction, and immunity). All these biological functions were previously identified in studies of signatures of selection on farmed rainbow trout [38] and other domesticated species [33, 39, 40, 116, 119, 120].

#### Hotspots of heterozygosity and balancing selection for fitness traits

In livestock species, many variants that are under balancing selection are known to improve zootechnical performances when present in the heterozygous state but to be deleterious in the homozygous state [139, 140]. In such cases, in general, only one homozygous state is lethal, while the alternative homozygous state is observed in the population (i.e. ROH can be identified). We identified four regions that are potentially involved in balancing selection and that lack long stretches of homozygosity. Although these regions display a high level of heterozygosity, the proteins encoded by the genes that they contain are highly conserved among vertebrates (see Additional file 2: Table S18). Many processes can explain these results that may be surprising at first glance. First, these regions may concentrate polymorphisms in non-coding parts of the genome. Polymorphisms in intronic regions of a gene can lead to different proteins via alternative splicing. In addition, there could be an excess of synonymous polymorphisms in the exons, without effects on proteins. Further analyses must be conducted to better understand the mechanisms that maintain such regions of extreme polymorphism, either validating the hypothesis of balancing selection or the existence of high mutation or recombination rates in these regions in the trout genome assembly.

In the heterozygous region chr10_a, the gene *ctnna2* (Table 7) plays an essential role in development of the brain in vertebrates [123]. In the Yunlong grouper, the *ctnna2* gene may be involved in vertebral development, since it is significantly differentially expressed between normal fish and fish with lordosis [124]. While the protein encoded by this gene is highly conserved among vertebrates ([123] and see Additional file 2: Table S18), its gene exhibits a high level of polymorphism in the four studied rainbow trout populations. However, a large part of this polymorphism is located in one of its introns (intron 6). In the Zebrafish Information Network (ZFIN) database, five transcripts have been identified for this gene (three mRNA and two non-coding RNA). We hypothesise that the polymorphism in the intronic region of *ctnna2* is essential for alternative splicing.

In the heterozygous region chr13_a (see Additional file 2: Table S17), the *map2k4* gene is involved in a variety of cellular processes (proliferation, differentiation, transcription regulation, development), plays a role in liver organogenesis and embryonic development during gastrulation, as demonstrated by morpholino-mediated knockdown in zebrafish [141], and is involved in immune response in the yellow catfish [142]. It has been suggested that the high degree of polymorphism of the *map2k4* gene is associated with the inflammatory process in immune response, which is consistent with our hypothesis of balancing selection and, more precisely, of a potential ancestral trans-species polymorphism in this genomic region [7, 143]. Trans-species polymorphism is a crucial evolutionary mechanism for sharing adaptative genetic variation across taxa [144]. The study of this mechanism has primarily concentrated on the genes of the major histocompatibility complex, but a few studies have described this process for other immune genes [145–147]. Maintaining genetic diversity in regions related to the immune system may be essential for resilience against various pathogens. In addition, this region on Omy13 has recently been identified as a significant quantitative trait locus (QTL) for resistance to high temperature [77].

In the heterozygous region chr19_a (Table 7), the gene *frem2* encodes an extracellular matrix protein that is required to maintain the integrity of skin and renal epithelia in zebrafish [128]. This protein is moderately conserved across vertebrates (see Additional file 2: Table S18). In a study on the detection of genomic regions with ancestral trans-species polymorphism shared between humans and chimpanzees [146], *frem*3, an important paralog of *frem2,* was identified under balancing selection. However, further studies should test the hypothesis of trans-species conservation of the *map2k4* and *frem2* genes to help decipher the various cellular processes in which they are involved.

### Hotspots of homozygosity and positive selection for essential biological functions

#### Regions and genes involved in early development

In the homozygous chr2_a and chr15_a regions, many genes play essential roles in early development and later in fitness (*cep162*, *tsnare1* and *mrap2* in the chr2_a, and *chn1*, *atp5mc1*, *zc3h15*, *nid2* and *brca2* in the chr15_a regions) (see Additional file 2: Table S12). In the homozygous chr2_b region (see Additional file 2: Table S12), the *pbx1* (*pre-B-cell leukemia transcription factor 1*) gene is related to early development in zebrafish [148] and was identified to be under positive selection in a Chilean farmed rainbow trout population [38]. Mutations in this gene generally cause major malformations, which have been suggested to play an essential role in survival in various species (zebrafish [148]; mouse [149]; and humans [150]).

Three genes located in close proximity in the chr16_a region between 46.42 and 46.53 Mb (see Additional file 2: Table S11), *samd10* (*sterile alpha motif domain-containing protein 10-like*), *dnajc5* (*dnaJ homolog subfamily C member 5-like*), and *tpd54* (*tumor protein D54*) were also detected to be in close proximity and under positive selection in ten modern goat breeds and one wild Bezoar goat breed [120]. This cluster of genes plays a role in survival and cellular processes (see Additional file 2: Table S12). In addition, in this chr16_a region, the *magi2* (*membrane-associated guanylate kinase, WW and PDZ domain-containing protein 2*) gene plays a vital role in the embryogenesis of zebrafish [151] and was identified to be under positive selection in a domesticated sheep breed compared to the wild Asiatic mouflon [115].

#### Regions and genes involved in neural and brain development and behaviour

In total, we identified seven genes that are primarily associated with neural and brain development in regions that were identified to be under positive selection (*tsnare1*, *cdk14*, *brsk2a*, *auts2*, *brd2, znf135*, and *grxcr1*). Some of these genes (*brsk2a*, *znf135*, *grxcr1*, *and auts2;* see Additional file 2: Table S12) may induce modifications of the behavior in farmed animals, which could be related to domestication processes [18, 118, 149, 152]. This is in line with results of Żarski et al. [153], who demonstrated that domestication modulates the expression of genes involved in neurogenesis. In particular, the *auts2* gene was previously identified to be under positive selection in cattle [122] and in domesticated Atlantic salmon populations from Canada and Scotland compared to their wild Atlantic salmon counterpart [116]. The *znf135* gene was also identified to be under positive selection in a farmed population of Atlantic salmon compared to a wild-type population [30]. These results strongly suggest that all these genes play a key role in domestication processes and may act on essential behaviors in both terrestrial and aquatic farmed animals.

#### Regions and genes involved in growth metabolism

Genes related to growth metabolism were present only in four regions that were identified to be under positive selection (see Additional file 2: Table S12), and none of these were present in regions of high heterozygosity. In the homozygous chr2_a region, the loss of function of the *mrap2* (*melanocortin-2 receptor accessory protein 2A*) gene is associated with severe obesity in many species (humans, zebrafish, rodent: [85]; sea lamprey: [86]; snakehead: [87]), and was shown to be under positive selection in the Chilean farmed rainbow trout population [38]. In addition, Yoshida et al. [154] identified this gene as a good candidate gene for a QTL associated with growth in Atlantic salmon. A QTL for sea lice resistance in rainbow trout [155] was also detected in the same region (between 10.43 and 11.81 Mb on the Swanson reference genome), which is presumably related to the interplay between resistance to sea lice, immune response and growth potential [156]. The homozygous chr2_b region includes two genes that were previously identified to be under positive selection, the *col9a2* (*collagen alpha-2(IX) chain*) gene, in a Scottish farmed population of Atlantic salmon [116], and the *scap* (*sterol regulatory element-binding protein cleavage-activating protein*) gene in six farmed Pacific white shrimp populations [117]. In the homozygous region chr2_d, the *igf-1α* (*insulin like growth factor receptor 1a*) gene has been shown to be differentially expressed between domesticated and wild populations of rainbow trout and coho salmon [157], and between larvae from domesticated spawners and larvae from wild spawners of the Eurasian perch [158]. In addition, the *igf-1* gene has been noted as a marker of domestication in dogs [118]. And finally, in the homozygous chr16_a region, the *emilin-3a* (*elastin microfibril interfacer 3a*) gene is known to be involved in muscle fiber development in zebrafish [159], and has been identified to be under positive selection in a F2 farmed population compared to the first generation (F1) of the domestication of a wild population of Australian snapper [40]. This signature of selection can, therefore, be considered as having resulted from domestication.

All identified growth-related genes are assumed to be associated with domestication. This assertion is confirmed for five genes (*mrap2*, *col9a2*, *scap*, *igf-1α*, and *emilin-3*) that were also identified to be under positive selection in various farmed populations (see Additional file 2: Table S12).

#### Regions and genes involved in reproduction

A few genes that are directly associated with reproduction were identified in highly homozygous regions (see Additional file 2: Table S12). In the homozygous chr2_b region, the *brd2* (*bromodomain-containing protein 2*) gene is involved in neural and brain development and in oogenesis and egg-to-embryo transition in zebrafish [160]. This gene was also identified to be under positive selection in a selected Canadian population of Atlantic salmon [116] and is located within a QTL for egg size in rainbow trout [161]. Khendek et al. [162] compared the reproductive performances (egg size, gonadal histology, hormonal levels) of Eurasian perch wild broodstock with those of domesticated and F1 fish and showed that domestication may have increased the diameter of the oocytes and the level of 17β-estradiol, and decreased the embryo survival of domesticated fish. In the homozygous chr15_a region, the *zp4* gene was identified to be under positive selection in a farmed Scottish population of Atlantic salmon compared to a wild population [116], and may be related to domestication.

#### Regions and genes involved in immunity

In farmed brown trout, Magris et al. [41] observed that regions that were identified to be under positive selection revealed an enrichment of Kyoto Encyclopedia of Genes and Genomes (KEGG) terms related to viral infection. However, in our study, only three genes related to immune function were detected and no enrichment of immune terms was observed in the GO analysis. The three immune function genes are located in a single region that was putatively under positive selection and in a single region that was identified to be under balancing selection. In the homozygous chr2_b region, two genes were related to immune functions, the *tnfaip8l2b* (*tumor necrosis factor, alpha-induced protein 8-like protein 2 B*) and *atg5* (*autophagy protein 5*) genes (see Additional file 2: Table S12). In the heterozygous chr16_a region, the *atp1b3* (*sodium/potassium-transporting ATPase subunit beta-1-interacting protein 3*) gene was identified to be under positive selection in farmed Atlantic salmon [119]. This gene induces NF-kappa B activation to inhibit viral replication, such as for hepatitis B, HIV, and EV71 [163, 164]. In addition, *atp1b3a* and *atp1b3b* paralogs have been hypothesized to be involved in the physiological response to low salinity in the Senegalese sole [165].

## Conclusions

We identified 13 regions under selection and these regions contained numerous genes that are involved in essential biological functions. By identifying signatures of selection that are shared by the four rainbow trout populations, we focused on regions related to ancient evolutionary processes that are essential for species survival. We identified only nine homozygous regions that are presumably under positive selection and four heterozygous regions that are putatively under balancing selection in all four populations. While shared homozygous regions may be associated with important biological functions that underly both fitness and domestication in rainbow trout, the heterozygous regions appeared to be mainly linked to fitness and survival functions (cell organization, embryonic development, and immunity) at different developmental stages or to functions involved in coping with various pathogens or abiotic stressors. Maintaining genetic diversity in these latter regions could be essential for species survival. This study confirmed the relevance of 17 genes that were previously identified to be under positive selection, 10 of which in fishes among other vertebrates (*auts2*, *atp1b3*, *zp4*, *znf135*, *igf-1α*, *brd2*, *col9a2*, *mrap2*, *pbx1* and *emilin-3*). We also identified new promising candidate genes that may be important for rainbow trout fitness. This study substantially increases our knowledge about the genomic location and nature of the genetic variation essential for fish survival. The candidate regions identified to be under selection and even more, those identified to be under balancing selection, are a material of choice for further investigation. Indeed, these results, in combination with new sequencing technologies that allow for long-fragment reads, will make it possible to better understand the fine genome dynamics involved in the selection process of such a complex genome.

### Supplementary Information

**Additional file 1: Figure S1.** Principal component analysis (PCA) of the genetic diversity of SY, and HA sub-populations (a) and of the five North American subpopulations grouped in the HA population (b), based on 546,903 SNPs. Elwha is the only wild population.

**Additional file 2:** **Table S1.** Shared signatures of selection between the French lines (LB, LC and SY) and the American pooled population (HA) detected by the XP-EHH statistics. **Table S2.** List of ROH islands for the LB population. **Table S3.** List of ROH islands for the LC population. **Table S4.** List of ROH islands for the SY population. **Table S5.** List of ROH islands for the HA population. **Table S6.** List of significant signatures of selection identified by iHS for the LB population. **Table S7.** List of significant signals of selection identified by iHS for the LC population. **Table S8.** List of significant signals of selection identified by iHS for the SY population. **Table S9.** List of significant signals of selection identified by iHS for the HA population. **Table S10.** iHS and ROH values per population within the nine overlapping regions detected among the four populations. **Table S11.** List of the 253 genes included in the nine shared genomic regions. In bold, genes identified as linked to domestication in the literature. **Table S12.** List and functions of the 17 genes annotated in the three homozygous regions (chr2_a, chr2_c and chr15_a) shared by the four rainbow trout populations, and the 15 genes in the six other regions previously identified in the literature as signatures of selection [159, 166–183]. **Table S13.** List of the heterozygous regions (i.e., large regions without ROH) for the LB population. **Table S14.** List of the heterozygous regions (i.e., large regions without ROH) for the LC population. **Table S15.** List of the heterozygous regions (i.e., large regions without ROH) for the SY population. **Table S16.** List of the heterozygous regions (i.e., large regions without ROH) for the HA population. **Table S17.** List of the 29 genes included in the four heterozygous genomic regions. **Table S18.** Percentage of protein homology between rainbow trout and nine other vertebrate species for the genes annotated in the heterozygous chr10_a, chr13_a, chr19_a and chr19_b regions.

## Data Availability

Restrictions apply to the availability of the data that support the findings of this study, which were used under license and are not publicly available. The data can be made available for reproduction of the results from Florence Phocas (florence.phocas@inrae.fr) on request via a material transfer agreement and with permission of the two breeding companies “Viviers de Sarrance” (Sarrance, France) and "Milin Nevez” (Plouigneau, France).
