# Supplementary figures and images for: Genome-wide detection of positive and balancing signatures of selection shared by four domesticated rainbow trout populations (Oncorhynchus mykiss)
Source: Genet Sel Evol. 2024 Feb 22;56:13. doi: 10.1186/s12711-024-00884-9 (PMC10882880; doi:10.1186/s12711-024-00884-9)

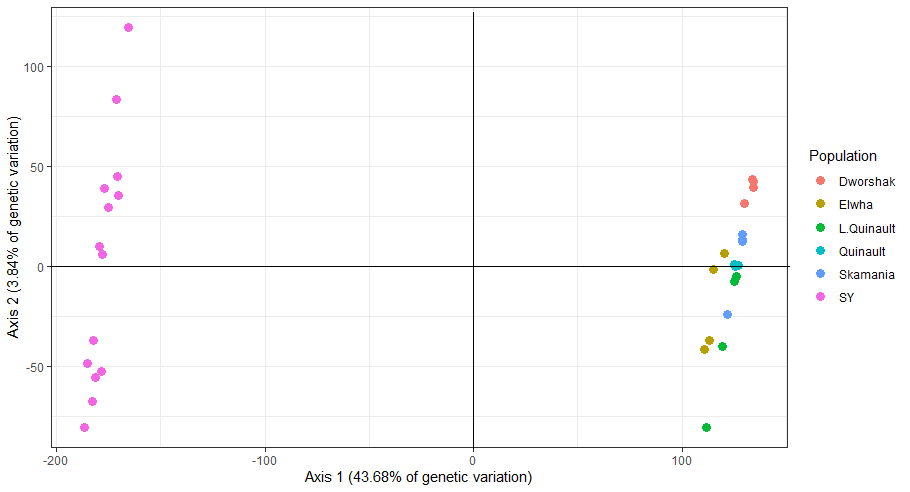


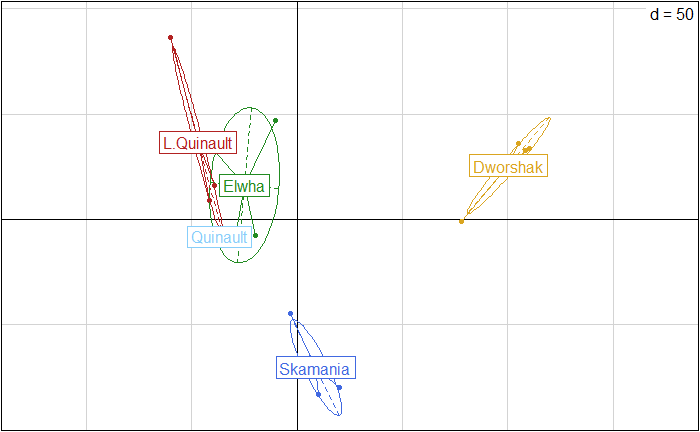


Axis 1 (12.99% of genetic variation)

Axis 2 (8.12% of genetic variation)

**(b)**

**(a)**

Supplement: Supplementary file 1 — Additional file 1: Figure S1. Principal component analysis (PCA) of the genetic diversity of SY, and HA sub-populations (a) and of the five North American subpopulations grouped in the HA population (b), based on 546,903 SNPs. Elwha is the only wild population. [file 12711_2024_884_MOESM1_ESM.docx]
